# AI trust and protean career among university students: the mediating role of intrinsic motivation and the moderating role of job insecurity

**DOI:** 10.3389/fpsyg.2025.1749655

**Published:** 2026-01-23

**Authors:** Juan Jiang, Jiachun Chen

**Affiliations:** Zhongshan Institute, University of Electronic Science and Technology of China, Zhongshan, China

**Keywords:** artificial intelligence trust, intrinsic motivation, job insecurity, protean career, university students

## Abstract

Amid the growing integration of artificial intelligence (AI) into the labor market, university students have to develop career orientation that aligns with the constant state of technological change. Drawing on self-determination theory and conservation of resources theory, we examine how AI trust influences university students' protean careers, while also considering the roles of intrinsic motivation and job insecurity. A survey of 576 undergraduates in Guangdong Province was conducted and analyzed using structural equation modeling. There are four key findings revealed by the results. First, AI trust significantly and positively influences university students' intrinsic motivation. Second, intrinsic motivation is positively associated with protean career. Third, intrinsic motivation mediates the relationship between AI trust and protean career. Fourth, job insecurity simultaneously intensifies the effect of AI trust on intrinsic motivation and enlarges the subsequent mediated path through which AI trust, via intrinsic motivation, translates into stronger protean career orientation. These findings shed new light on the psychological micro-behavior mechanisms by which technological beliefs influence career attitudes, and offer pragmatically useful implications for AI-related education programs, university career counseling, corporate personnel management, and the self-development of students.

## 1 Introduction

The rapid advancement of artificial intelligence (AI) is profoundly reshaping the structure of the global labor market and individual career trajectories (Li K. et al., 2025). As a cohort at the forefront of technological transformation, university students are continuously exposed to the direct impacts of AI (Chen et al., 2025). AI is not merely an external environmental factor but has become a critical determinant of their career choices and planning (Presbitero and Teng-Calleja, 2023). On one hand, AI-driven skill iteration compels students to constantly adjust their career paths to meet evolving job demands (Parent-Rocheleau et al., 2024; Parker and Grote, 2022), rendering traditional stable career trajectories increasingly fragile (Brougham and Haar, 2018; Kong et al., 2021). In this context, the protean career—characterized by self-direction and values-drivenness—has arisen as a core strategy for navigating career uncertainty (Briscoe and Hall, 2006). On the other hand, AI also offers powerful tools for career exploration (Eren et al., 2025). If students maintain confidence in artificial intelligence, the efficiency of these tools can effectively accelerate career development. AI trust is defined as an individual's positive attitude toward AI and recognition of the possible impact of AI on their roles and responsibilities (Glikson and Woolley, 2020; Kong et al., 2021). When students view AI as reliable, useful, and valuable, they actively use it to navigate career uncertainty (Kong et al., 2023), and the utilization of AI has an impact on their career choices. Therefore, the trust of AI is the basic connection node between the technology of AI and career thinking of students, and it is the central entrance to understanding the evolution of career orientations in the digital era.

The historical concept of careers in stable organizations is no longer fit for the AI-driven labor market (Brougham and Haar, 2018; Kong et al., 2021). Instead, university students increasingly develop protean career that is focused on self-direction and values individual growth rather than organizational allegiance (Briscoe and Hall, 2006). Because this attitude encourages flexibility and initiative, it helps students to deal with uncertainties in employment (Chui et al., 2022). By designing their own career routes and being self-taught on new skills (Huang et al., 2024), people minimize the risk of AI-based job loss and align their jobs with their personal values (Chen et al., 2025). However, protean career is not only a matter of individual traits but also is dependent on environmental pressures like social background and labor market conditions (Rodrigues et al., 2019). In the case of distrust in AI, students feel threatened technically and establish professional anxiety (Chen et al., 2025) and are not able to create the route proactively. High AI trust, on the other hand, alleviates this fear and persuades people that AI could potentially improve work and life (Kong et al., 2023), thus triggering the development of protean career. Investigating the link between AI trust and protean career is therefore vital for satisfying students' growing career-development needs.

Current research demonstrates three important gaps. First, most studies analyze AI's macro-level implications on the labor market (Frey and Osborne, 2017), such as job creation or skill shifts, or they link AI cognition and AI usage to career attitudes (Presbitero and Teng-Calleja, 2023). These investigations overlook AI trust, a deeper subjective variable (Kong et al., 2023). AI cognition just communicates awareness, and AI usage can be driven by external pressure, whereas AI trust shows the basic notion that AI is reliable and good, this belief is the crucial precondition for technology to impact career direction (Hoff and Bashir, 2015). However, it has received limited attention (Kong et al., 2021). Second, antecedent research on university students' protean career focuses on traditional elements such as individual attributes, family factors, and social milieu (Rodrigues et al., 2019). It mostly ignores whether developing technologies influence this orientation. Therefore, it cannot explain how protean career evolves with technological change. Third, although a few studies identify a connection between AI and protean careers (Kong et al., 2023), they do not deconstruct the internal mechanism by which AI trust influences protean career. As a result, our comprehension of the AI trust–protean career relationship remains rudimentary.

To cover the foregoing gaps, our research questions are as follows: Does university students' AI trust influence their protean career? If yes, how does this effect occur and under which conditions?

To explain how AI trust influences university students' protean career, we integrate self-determination theory (SDT) with conservation of resources theory (COR). SDT claims that intrinsic motivation relates external conditions to individual conduct, and this motivation is engaged when competence and autonomy demands are met (Deci and Ryan, 1980, 2008). AI trust can satisfy these two needs. When students believe that AI technologies are reliable and useful, they feel more capable and autonomous in career preparation. This idea makes people want to deal with job uncertainty on their own, which leads to the self-directed, values-driven mindset that is characteristic of protean career (Briscoe and Hall, 2006).

COR theory suggests that people attempt to safeguard existing resources and gain new ones to offset the potential of resource loss (Hobfoll, 1989). Job insecurity represents the perceived threat of losing professional resources, it is characterized as the dread of involuntary job loss and the worry that one's position may lack long-term stability or that career trajectories may be altered (Greenhalgh and Rosenblatt, 1984; Witte, 1999). Despite not yet entering the formal labor market, university students are highly aware of the potential threats that AI poses to future job stability and career opportunities (Chen et al., 2025). Therefore, applying the concept of job insecurity to university students is justified, as it effectively captures their psychological reactions and anxieties regarding future career uncertainties. Moreover, this perception not only influences their current learning motivation and career planning but may also extend to future career choices and adaptability (Prentice et al., 2023). Empirical research reveals that work instability moderates how individual qualities and environmental circumstances shape employee attitudes and behavior (Chen et al., 2024). From a COR perspective, when individuals feel impending resource loss, they immediately seek external help that can replace resources and relieve distress (Hobfoll et al., 2018). AI trust—an assumption that AI can give professional resources—represents such support (Kong et al., 2023). Therefore, when job insecurity is severe, students' anticipation of resource replenishment from AI grows higher, and the favorable effect of high AI trust on intrinsic motivation to update skills and handle uncertainty is reinforced (Eren et al., 2025). In short, job insecurity may condition the relationship between AI trust and intrinsic motivation.

Our study makes three contributions. First, it ties AI trust to university students' protean career. By centring on AI trust as a key subjective predictor, and by integrating self-determination theory with conservation of resources theory, we specify how AI trust shapes protean career and enrich research on factors that drive career orientations under new technologies. Second, we refine the mechanism. We build a comprehensive framework that positions intrinsic motivation as the mediator between AI trust and protean career, and job insecurity as the moderator of the AI trust–intrinsic motivation link. This framework shows the comprehensive path from AI trust to protean career and gives us a systematic way to think about how career orientation is formed in the digital age. Third, we strengthen practical guidance. For students, we clarify that building positive AI trust and activating intrinsic growth motives are essential for developing an AI-era protean career, reducing uncertainty anxiety, and proactively adapting to labor-market shifts. For universities, we provide direction for career counseling and AI education. Hands-on AI experiences can foster rational trust, while counseling that considers students' job-insecurity levels can deliver targeted psychological support and career planning, helping students cultivate flexible career. For organizations, we underscore that they can enhance talent adaptability and mutual growth by integrating AI trust and protean career into recruitment and development practices.

## 2 Theoretical analysis and research hypotheses

### 2.1 AI trust and intrinsic motivation

SDT posits that intrinsic motivation is sparked and sustained when three basic psychological needs are met: competence (the belief that one can reach the goal), autonomy (the sense that one can choose the direction and make decisions), and relatedness (the feeling of being meaningfully connected to the environment and others) (Deci and Ryan, 1980). Intrinsic motivation is an internal driving factor that originates from love and passion, which has nothing to do with remuneration and profit (Liang et al., 2022). When external circumstances such as technical tools or social supports satisfy these demands, intrinsic motivation is awakened and promotes active, persistent goal-related behavior (Deci and Ryan, 2008). In this study, AI trust—students' positive perception that AI is dependable and valuable—functions as such a supportive external factor (Kong et al., 2023; Lin et al., 2025). Intrinsic motivation is the inner desire to overcome AI-induced employment insecurity by actively developing skills and achieving self-fulfillment (Liang et al., 2022). A favorable belief in technology supports an open attitude toward new technology and inspires individuals to learn the skills required to use it effectively (Suseno et al., 2022). Specifically, we propose that AI trust enhances students' intrinsic motivation by satisfying their needs for competence and autonomy.

AI trust fosters intrinsic motivation by fulfilling the need for competence. The need for competence, emphasizes the belief in one's capacity to tackle challenges and achieve desired outcomes, is a cornerstone of intrinsic motivation (Deci and Ryan, 2008). AI trust satisfies university students' competency need through two approaches. First, the tool value of AI lowers the bar for skill acquisition (Chen et al., 2025). Students with high AI trust view AI as a powerful enabler for learning and task execution (Kong et al., 2023) and believe that it can handle ordinary work (Mirbabaie et al., 2022) to liberate them to focus on developing higher-order core capabilities (Zirar et al., 2023). AI also acts as a learning tool that helps them learn relevant career-learning skills quickly (Chowdhury et al., 2022), thus overcoming the worry about being under-qualified and inducing the intrinsic motivation to learn better. Second, the reliability of AI gives a higher sense of control. Competence stems not just from having tools, but from having reliable tools. AI nowadays drives an enormous range of fast-growing economic activities and is often more reliable than human beings (Jia et al., 2024). Reliability is the basis of trust (Arslan et al., 2022), and high trust in AI implies a belief in its stability and dependability (Glikson and Woolley, 2020). This belief provides students with a sense that their efforts to upskill with AI will not be thwarted by technical failures, reinforcing a feeling of control that “I can improve through my own effort” (Toma and Hudea, 2024). This perceived control is a fundamental psychological source of intrinsic motivation.

AI trust nurtures intrinsic motivation by satisfying the need for autonomy. Autonomy need-the idea that acts and decisions come from human volition and not from external coercion-is a another characteristic of self-determination theory that facilitates intrinsic motivation (Deci and Ryan, 1980). AI trust fulfills the need for autonomy of university students by enabling independent decisions regarding career choices. First, it breaks down traditional external barriers to career exploration (Brougham and Haar, 2018). Historically, the development of skills, and career exploration are usually limited by the availability of resources, with little scope left for autonomous choice (Briscoe and Hall, 2006). High AI trust signals recognition of AI's “boundaryless support” (Kong et al., 2021). Students believe AI can give varied learning resources (Chen et al., 2025) and open multiple job pathways, freeing them from external limits. They think that they can pick what to learn, and which directions to pursue (Gerçek and Özveren, 2025). Second, it reinforces the student's agency in human-AI collaboration. High AI trust does not entail outsourcing decisions to AI, rather, it demonstrates confidence that AI will support personal goals (Jia et al., 2024). In this “human-led, AI-assisted” paradigm, students remain the primary agents, deciding how and when to use AI to advance their ambitions (Chu et al., 2025). This “leader” role perception generates a strong sense of autonomous control and promotes the intrinsic motivation to set objectives and explore jobs independently. Thus, we propose:

**Hypothesis 1: AI trust will be positively related to university students' intrinsic motivation**.

### 2.2 Intrinsic motivation and protean career

SDT claims that intrinsic motivation is a psychological drive rooted in personal interest, growth demands, and self-fulfillment goals. Its essential features are initiative and goal-directedness (Deci and Ryan, 2000). A protean career is characterized by two core dimensions: self-direction and valuing orientation (Briscoe and Hall, 2006). We argue that intrinsic motivation serves as the psychological engine for both dimensions. When intrinsic motivation is activated, people are more ready to pass through the bounds set by the outside world, actively pursue routes that meet their needs for development, and maintain these behaviors over the long term without being seduced by immediate gains or pressures (Deci and Ryan, 2008).

An important component of protean career is autonomous motivation in career development (Briscoe et al., 2006), and intrinsic motivation is the psychological drive behind this autonomy. With regard to SDT, intrinsic motivation involves the satisfaction of the need for autonomy (Deci and Ryan, 1980). When students are focused through intrinsic motivations, including the need to test or improve skills or attain self-fulfillment (Gerçek and Özveren, 2025), they view their work as a means to achieve self-actualization instead of simply a means to make a living (Jackson and Tomlinson, 2019). This attitude creates a strong drive for self-earning. Intrinsic motivation is driven when a student self-activates in making different career choices that can make them more competitive and will also help them to achieve personal goals and objectives (Hirschi and Koen, 2021). They are no longer waiting for universities or employers to tell them how. They autonomously determine the necessary competences and acquire the corresponding experience on their own through learning and performing (Chen et al., 2025), which results in a self-guided logic of career, which is considered as the autonomous control component of protean career. Technology keeps the labor market in constant motion (James and Cardador, 2007), Students who are high in intrinsic motivation view this flux as a growing opportunity, rather than a threat. When career adjustments or skill updates are needed, they take the initiative—learning across disciplines, seeking new opportunities—to protect their autonomy. This pattern of “active adaptation to change” is a feature of protean career.

The second fundamental aspect of protean career is the adoption of one's own values as the major criterion for career decisions (Briscoe et al., 2006). Intrinsic motivation helps students identify what they value and then translates these values into the guiding rule of their career (Jackson and Tomlinson, 2019). Self-determination theory suggests that intrinsic motivation is primarily the pursuit of self-fulfillment (Deci and Ryan, 2008). This goal causes individuals to measure employment possibilities against personal progress and self-realization rather than against traditional external signals such as money or job title. Students who are intrinsically motivated pick employment that offers skill advancement and new experiences even if these positions are tough or pay less in the near run (Bravo et al., 2017; Chui et al., 2022). When they hunt for internships or full-time work, they choose roles that expose them to AI technologies and new initiatives. This competence-growth logic provides an orientation that is separated from conventional standards and centered on personal needs. For these students, career growth is a self-realization process that must meet their interests and values (Cheng and Nguyen, 2024). If the current environment fails to satisfy these needs—because growth is constrained or values clash—people initiate change: they switch tracks or create new possibilities rather than accept the status quo (Choi et al., 2013). This self-fulfillment logic is exactly what distinguishes protean career from standard career attitudes (Briscoe and Hall, 2006). Thus, we propose:

**Hypothesis 2: Intrinsic motivation will be positively associated with university students' protean career**.

### 2.3 The mediating effect of intrinsic motivation

AI trust satisfies both the competence and autonomy needs of university students. On the one hand, high AI trust encourages students to see the tool value and reliability of AI, convincing them that AI may assist in upgrading the skills required to cope with professional uncertainty, and so alleviating anxiety about perceived inadequacies (Chen et al., 2025). On the other side, high AI trust convinces students that they can determine for themselves how AI will be deployed to fulfill their career goals, liberating them from external resource limitations (Chu et al., 2025). The realization of these two requirements directly arouses an intrinsic motivation to increase abilities and overcome job uncertainties. Intrinsic motivation is the psychological force that propels students to pursue, self-growth, and value realization (Jackson and Tomlinson, 2019). Once this desire is awakened, students consider professional advancement as a route for self-fulfillment. They improve their sense of autonomous control, cease to rely on external arrangements, and actively plan career pathways, and respond to labor-market changes (Chui et al., 2022). At the same time, they make competence growth, and value congruence the major criteria in professional decisions, favoring directions that satisfy developmental requirements even if these choices transcend traditional job limits (Choi et al., 2013). This combination of autonomous control, and self-demand-oriented cognition, and action represents the basic attribute of protean career (Briscoe and Hall, 2006). Intrinsic motivation, therefore, transmits the influence of AI trust to protean orientation. Thus, we propose:

**Hypothesis 3: Intrinsic motivation will mediate the relationship between AI trust and university students' protean career orientation**.

### 2.4 The moderating role of job insecurity

The main concept of COR theory is that humans are innately driven to preserve existing resources, and acquire new ones in anticipation of probable resource loss (Hobfoll, 1989). When people sense a fear of future resource depletion, they feel resource-loss anxiety (Hobfoll et al., 2018) and become much more reliant on any resource that can alleviate this anxiety (Halbesleben et al., 2014).

According to COR theory, persons who possess abundant resources are less influenced by the stress of resource loss (Hobfoll, 2002). The antithesis of job insecurity is job security, defined as employees' trust in their future position within the business (Kuhnert and Palmer, 1991). Job security can be seen as a vital personal psychological resource for employees. In the context of rapid AI advancement, which has been shown to displace certain human occupations and pose a tangible threat to the workforce (Prentice et al., 2023), job security becomes particularly vital. It gives a sense of control and internal regulation, hence boosting employees' work engagement and performance (Kraimer et al., 2005). Moreover, job security instills faith that technology will augment rather than replace their labor (Prentice et al., 2023). Therefore, when individuals feel high work insecurity, it suggests they lack this essential psychological resource of job security, making them more susceptible to behavioral pressures.

Although university students have not yet entered the formal labor market, they are fully aware of AI's impact on occupations (Chen et al., 2025). When job insecurity is low, individuals perceive a minimal risk of future resource loss and thus experience only mild resource-loss anxiety. Under such conditions, they report lower levels of stress, and uncertainty, along with a stronger sense of control over their work and life (Hsieh and Huang, 2017). Given the low urgency to enhance career-related resources through external means, such as AI, students lack the impetus to translate their positive trust in AI into active, skill-building motivation. Some may even assume their future career paths are relatively secure and hence perceive no immediate need to leverage AI for competency development. As a result, the positive effect of AI trust on intrinsic motivation remains limited, and the association between the two variables is weakened.

In contrast, job insecurity fundamentally reflects a sense of uncertainty and ambiguity regarding one's occupational future (Sverke and Hellgren, 2002). In highly uncertain, and ambiguous contexts, individuals experience diminished control over their work and life, leading to a loss of perceived mastery (Hsieh and Huang, 2017). Job insecurity leads to resource loss (Chen et al., 2024). When university students perceive significant employment uncertainty, they become acutely aware that fundamental professional resources such as job stability, and development chances may be lost, and resource-loss anxiety escalates. According to COR, students would urgently seek external supports that can restore career resources, and ease loss anxiety (Chui et al., 2022). AI trust perfectly matches this support: high AI trust means students believe AI is a reliable resource-replenishment tool that may help them upgrade essential abilities to enhance employability, and explore new career possibilities. Driven by anxiety-induced resource demands, students are more likely to convert their trust in AI into intrinsic motivation to develop skills, and navigate job uncertainties, thereby significantly strengthening the positive effect of AI trust on intrinsic motivation. In other words, the strength of this link will be much stronger under high rather than low job insecurity. Therefore, we propose:

**Hypothesis 4-1: Job insecurity moderates the relationship between AI trust and intrinsic motivation, such that the positive effect of AI trust on intrinsic motivation is stronger when job insecurity is high**.

The above analysis suggests that when university students perceive high job insecurity, they are more inclined to view trust in AI as a crucial means of replenishing career resources and relieving anxiety, leading them to redouble their efforts in skill building, and self-directed career exploration. This anxiety-driven search for resources makes the effect of AI trust on intrinsic motivation more pronounced and further amplifies the translation of that motivation into a protean career. In other words, job insecurity not only moderates the relationship between AI trust and intrinsic motivation, but also creates a step-by-step amplification effect throughout the sequential process by which trust influences orientation through motivation. Thus, we propose:

**Hypothesis 4-2: Job insecurity significantly moderates the mediated effect of AI trust on protean career via intrinsic motivation, such that the mediation becomes stronger when job insecurity is high**.

**Figure 1:**
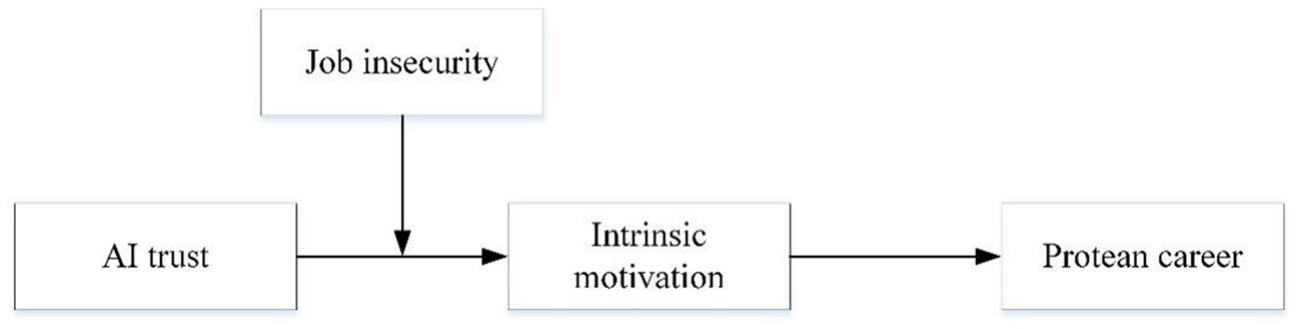
Research model.

Our research model is shown in Figure 1.

## 3 Method

### 3.1 Sample and data collection

The questionnaire was administered from 22 February to 15 March 2025. An electronic poll was constructed on Wenjuanxing and distributed by convenience, and snowball sampling. Invitations were placed in course WeChat groups, student clubs, libraries, and study rooms across more than 80 universities in Guangdong Province. Students were assured that participation was voluntary. To ensure data quality, the following exclusion criteria were applied when screening questionnaires: (1) Responses with obvious careless filling patterns, such as identical ratings for all items (e.g., selecting “3 = neutral” for every question) or consecutive identical answers without logical variation; (2) Incomplete questionnaires with more than 10% of items unanswered; (3) Responses completed in an unreasonably short time (less than 60 seconds for the full questionnaire); (4) Duplicate responses identified by overlapping IP addresses or identical personal information (e.g., age, grade) from the same respondent. A total of 700 questionnaires were distributed, 576 valid replies were returned, producing an effective response rate of 82.3%. Sample composition: 39% male, 61% female. The sample spans all undergraduate years: 23% freshmen, 46% sophomores, 21% juniors, and 10% seniors. The distribution across disciplines is balanced, with 50% of participants majoring in STEM fields and the remaining 50% in humanities or social sciences. The survey covers a wide spectrum of higher-education institutions across Guangdong Province, including both comprehensive research universities and application-oriented colleges. Specifically, the sample sizes of respondents from representative universities are as follows: Sun Yat-sen University (44), South China University of Technology (24), Jinan University (19), Guangdong University of Technology (33), Guangdong University of Finance (40), University of Electronic Science and Technology of China, Zhongshan Institute (97), Guangzhou College of Applied Science and Technology (25), and Guangzhou Institute of Technology (16). This multi-level sampling strategy ensures the good representativeness of the sample.

### 3.2 Measurement

All key constructs were examined with standard measures. Wording was partially adapted for university students by substituting “job/work” with “future job/work.” All items use a 5-point Likert scale (1 = strongly disagree, 5 = strongly agree).

AI trust: Eleven-item measure from Kong et al. (2023). Sample items: “I believe AI technology can facilitate routine, and trivial tasks through automation”; “I believe AI adoption won't reduce the focus on human skills such as creative intellect in my future job.”

Intrinsic motivation: Six-item measure modified from Liang et al. (2022) that focuses on growth demands, and self-realization prompted by AI uncertainty. Sample items: “To overcome the uncertainty brought about by AI, I hope that my future work will provide me with the opportunity to increase my knowledge and abilities.”; “Overcoming the uncertainty brought about by AI helps me with self-expression.”

Protean career: a fourteen-item scale developed from Briscoe et al. (2006) questionnaire, which covers two dimensions: Self-directed (8 items) and Values-driven (6 items). Samples: “I am in charge of my own career”; “It doesn't matter much to me how other people evaluate the choices I make in my career.”

Job insecurity: Five-item measure from He et al. (2024) which incorporates worries about future stability and opportunity loss. Sample items: “I am worried about the possibility of losing my job in the future.”; “I think my future job is likely to change.”

We included age, gender, and grade as control variables based on theoretical and empirical justifications in career research. First, gender may correlate with career orientation, and technology attitudes: prior studies have shown that female and male students often differ in their perceived relevance of AI to career development (Chen et al., 2025), and willingness to adopt self-directed career strategies (Rodrigues et al., 2019). Controlling for gender helps rule out confounding effects from gender-related differences in career perceptions. Second, age and grade reflect students' career maturity and exposure to labor-market information: senior students or older undergraduates typically have more internship experience and clearer career expectations (Huang et al., 2024) which may independently influence their intrinsic motivation and protean career orientation. By controlling these variables, we isolate the unique effects of AI trust, intrinsic motivation, and job insecurity on the focal relationship.

### 3.3 Data analysis strategy

In our study, to examine the relationships among university students' trust in AI, intrinsic motivation, and protean career, we opted for Partial Least Squares Structural Equation Modeling (PLS-SEM) as our analytical approach. PLS-SEM is particularly suited for our research at this stage of theoretical development, as it does not require a fully confirmed measurement model, and provides reliable estimates even with a moderate sample size (*n* = 576). Moreover, PLS-SEM is capable of effectively handling complex models that include multiple mediators and moderators, and it emphasizes the predictive power of the model, which is crucial for understanding the practical implications of our findings. Explanatory power is satisfactory: *R*^2^ = 0.431 for intrinsic motivation and 0.522 for protean career. Every structural path shows at least a medium effect (*f*^2^ ≥ 0.14), and Q^2^ > 0, confirming predictive relevance. Multicollinearity is not a concern: all item-level VIFs are below 3, well below the conventional threshold of 5, indicating that predictors are not highly inter-correlated. These analyses collectively provide strong statistical support for our model.

## 4 Results

### 4.1 Reliability and validity

Table 1 reveals that Cronbach's α for every scale is above 0.80 and composite reliability (CR) is above 0.88, suggesting good internal consistency. With the exception of protean career (AVE = 0.498, just below the 0.50 standard), the average variance extracted (AVE) for all other components is larger than 0.50, showing satisfactory convergent validity. As Briscoe and Hall (2006) consistently argue in their scale-development work, the protean career is not a unidimensional trait but a higher-order construct constituted by two related yet distinct facets—self-directed and values-driven. Adhering to this theoretical specification, we modeled the two facets as first-order factors that reflect a second-order protean-career factor. The resulting AVE for the second-order construct is 0.498, marginally below the 0.50 threshold; however, the AVEs of the first-order dimensions are 0.547 and 0.553, showing satisfactory convergent validity. Briscoe et al. emphasize that only when both facets are present does an individual truly exhibits a “self-directed, values-driven” protean attitude, so the higher-order structure is theoretically presupposed. Empirically, the composite reliability of 0.933 further confirms that overall measurement precision is not compromised by the slight AVE drop. Consequently, the marginal AVE is acceptable under the dual guarantee of theoretical grounding, and empirical quality.

**Table 1:** Construct reliability and validity.

**Variables**	**Cronbach's alpha**	**Composite reliability**	**Average variance extracted (AVE)**
AI trust	0.915	0.928	0.544
Job insecurity	0.88	0.907	0.662
Intrinsic motivation	0.88	0.909	0.624
Self-directed	0.881	0.906	0.547
Values-driven	0.838	0.881	0.553
Protean career	0.922	0.933	0.498

*n* = 576.

Table 2 presents the results of the discriminant validity analysis using the Heterotrait-Monotrait (HTMT) method. The HTMT ratio is a technique for assessing the discriminant validity of constructs by comparing the average inter-construct correlation with the average intra-construct correlation. According to the data in the table, all HTMT ratios between the construct pairs are below 0.90, and the 95% confidence intervals do not include 1, indicating good discriminant validity among the constructs. Specifically, the HTMT ratio between AI trust and job insecurity is 0.274, with a 95% confidence interval of (0.185, 0.38); between AI trust and intrinsic motivation, it is 0.69, with a 95% confidence interval of (0.608, 0.768); and between AI trust and protean career, it is 0.695, with a 95% confidence interval of (0.611, 0.772). These results suggest that while these constructs may theoretically be related, they are distinctly measured, thus supporting the discriminant validity of the model.

**Table 2:** Discriminant validity (HTMT).

**Variables**	**AI trust**	**job insecurity**	**intrinsic motivation**
AI trust			
Job insecurity	0.274 (0.185,0.38)		
Intrinsic motivation	0.69 (0.608,0.768)	0.175 (0.107,0.292)	
Protean career	0.695 (0.611,0.772)	0.287 (0.185,0.393)	0.725 (0.655,0.787)

*n* = 576; the numbers in parentheses are the 95% confidence intervals.

### 4.2 Descriptive statistics

Table 3 shows the descriptive statistics and correlations. AI trust has a mean of 3.548 (SD = 0.727), demonstrating a reasonably high level of trust in AI among university students with notable individual variation. Job insecurity averages 3.514 (SD = 0.866), suggesting a reasonably strong concern about future employment stability and a relatively large variety of scores. Intrinsic motivation records a mean of 3.568 (SD = 0.783), demonstrating that students generally experience a strong internal urge to grow in response to AI-induced job uncertainty. Protean career averages 3.594 (SD = 0.673), suggesting that students overall embrace a self-directed, values-driven approach to career development.

**Table 3:** Descriptive statistics.

**Variables**	**Mean**	**S.d**.	**1**	**2**	**3**	**4**	**5**	**6**	**7**
1. AI Trust	3.548	0.727	1						
2. Job insecurity	3.514	0.866	0.237^**^	1					
3. Intrinsic motivation	3.568	0.783	0.618^**^	0.149^**^	1				
4. Protean career	3.594	0.673	0.638^**^	0.255^**^	0.653^**^	1			
5. Gender	0.387	0.488	−0.029	−0.117^**^	0.04	0.033	1		
6. Age	19.972	1.251	0.051	0.003	−0.01	0.024	0.109	1	
7. Grade	2.179	0.899	0.035	−0.033	−0.026	0.021	0.108	0.636^**^	1

*n* = 576; ^**^p < 0.01.

Among the correlations, AI trust and intrinsic motivation are positively connected (*r* = 0.618, *p* < 0.01), providing initial support for Hypothesis 1. Intrinsic motivation and protean career are likewise favorably linked (*r* = 0.653, *p* < 0.01), suggesting preliminary evidence for Hypothesis 2.

### 4.3 Model results

We tested the research model with SmartPLS (Table 4). The results show that AI trust exerts a significant positive effect on intrinsic motivation (Estimate = 0.625, SE = 0.042, *t* = 14.833, *p* < 0.001), supporting Hypothesis 1. Intrinsic motivation, in turn, significantly predicts protean career (Estimate = 0.419, SE = 0.051, *t* = 8.222, *p* < 0.001), supporting Hypothesis 2.

**Table 4:** Model results.

**Path**	**Estimate**	**SE**	***T* statistics**	***P* values**
AI trust → intrinsic motivation	0.625	0.042	14.833	0.000
AI trust → protean career	0.383	0.056	6.79	0.000
Intrinsic motivation → protean career	0.419	0.051	8.222	0.000
Job insecurity → intrinsic motivation	0.019	0.041	0.453	0.650
Job insecurity x AI trust → intrinsic motivation	0.15	0.047	3.228	0.001
Age → protean career	−0.009	0.045	0.195	0.845
Gender → protean career	0.067	0.062	1.078	0.281
Grade → protean career	0.022	0.042	0.532	0.595

*n* = 576.

Using the bootstrap procedure with 5,000 resamples (Table 5), we found that intrinsic motivation exerts a significant indirect effect between AI trust, and protean career (Estimate = 0.262, 95% CI [0.193, 0.328], which does not include zero), confirming the mediation effect and supporting Hypothesis 3.

**Table 5:** Indirect effects.

**Path**	**Estimate**	**2.5%CI**	**97.5% CI**
AI trust → intrinsic motivation → protean career	0.262	0.193	0.328
Job insecurity → intrinsic motivation → protean career	0.008	−0.021	0.047
Job insecurity x AI trust → intrinsic motivation → protean career	0.063	0.029	0.102

*n* = 576.

**Figure 2:**
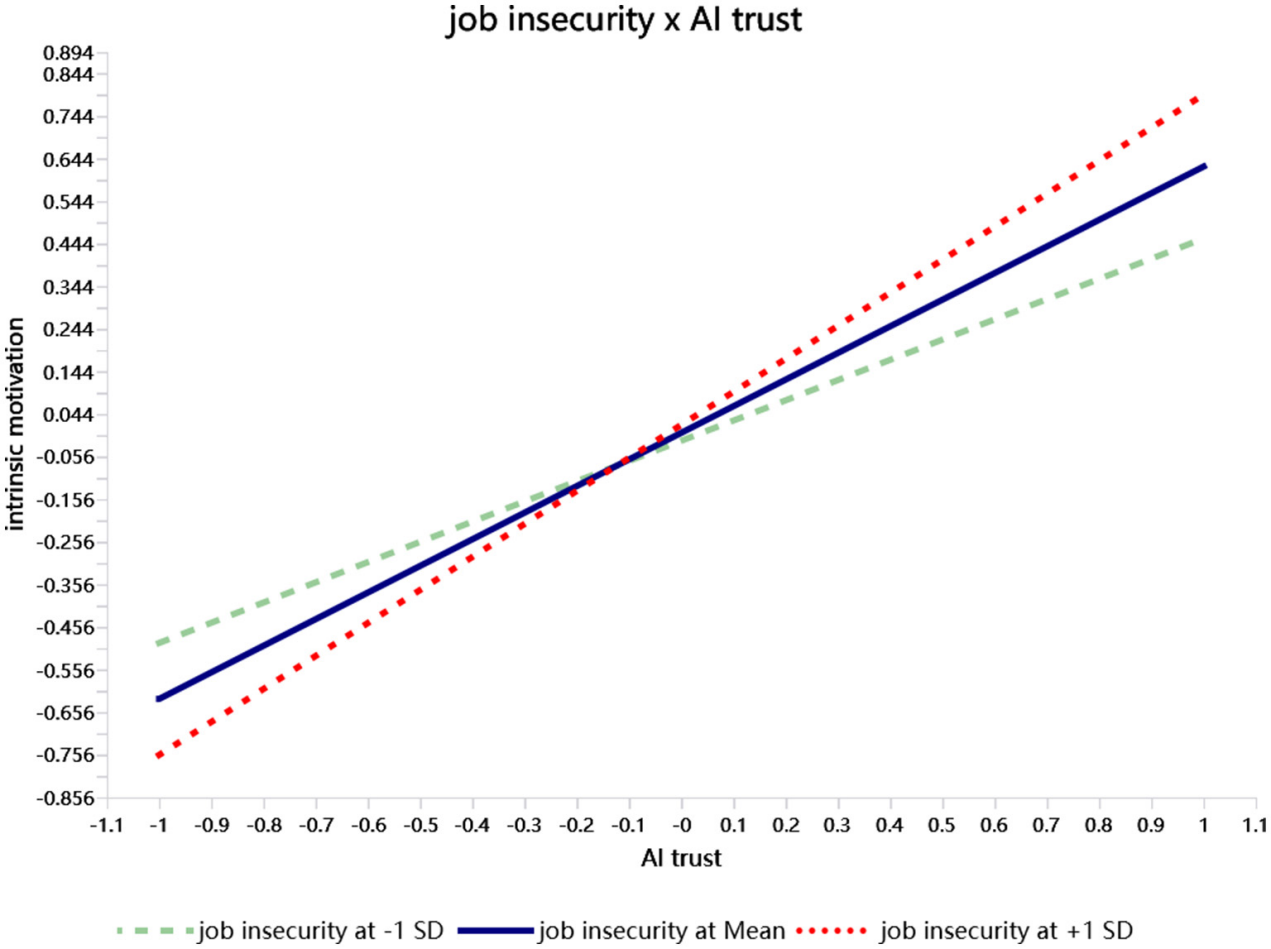
Simple slope analysis.

The moderated-effect results (Table 4) show that the interaction term between AI trust and job insecurity is significantly and positively related to intrinsic motivation (Estimate = 0.150, SE = 0.047, *t* = 3.228, *p* < 0.01). Simple-slope tests (Figure 2) reveal that the positive effect of AI trust on intrinsic motivation is stronger when job insecurity is high (M + 1 SD) and weaker when job insecurity is low (M – 1 SD). These findings confirm that job insecurity significantly amplifies the relationship between AI trust and intrinsic motivation, supporting Hypothesis 4-1.

The results in Table 6 show that the indirect effect of AI trust on protean career through intrinsic motivation is significantly stronger under high job insecurity (Effect = 0.325) than under low job insecurity (Effect = 0.199). The moderated mediation index is 0.063 (95% CI does not include 0), indicating that job insecurity significantly strengthens the mediating role of intrinsic motivation in the relationship between AI trust and protean career. Hypothesis 4-2 is supported.

**Table 6:** Moderated mediation.

**Path**	**Moderator**	**Effect**	**SE**	**2.5%CI**	**97.5%CI**
AI trust → intrinsic motivation → protean career	Job insecurity at +1SD	0.325	0.038	0.247	0.395
	Job insecurity at −1SD	0.199	0.041	0.121	0.279
	Job insecurity at mean	0.262	0.035	0.193	0.328
	Moderated mediation index	0.063	0.019	0.029	0.102

*n* = 576.

### 4.4 Common method bias

To assess common method bias (CMB), we conducted Harman's single-factor test. Four factors emerged with eigenvalues greater than 1, and the first factor explained 36.59% of the total variance, well below the 50% threshold. Thus, common method bias is not a concern in this study.

In addition, to test for potential CMB, we conducted a confirmatory factor analysis (CFA) with a single factor, loading all self-reported items onto one underlying factor. The results (Table 7) indicated that, compared to the original model, the fit indices of the single-factor model were significantly poorer, with GFI = 0.61, CFI = 0.675, NFI = 0.641, IFI = 0.676, TLI = 0.655, and RMSEA = 0.102. These findings suggest that the single-factor model did not fit the data well, thereby supporting the conclusion that the data in this study are not significantly affected by CMB.

**Table 7:** Confirmatory factor analysis.

**Model**	**GFI**	**CFI**	**NFI**	**IFI**	**TLI**	**RMSEA**
Original model	0.884	0.931	0.884	0.931	0.925	0.048
One-factor model	0.61	0.675	0.641	0.676	0.655	0.102

## 5 Discussion

### 5.1 Main findings

We discover that AI trust greatly enhances university students' intrinsic motivation, a result that accords closely with the logic of competence-need and autonomy-need satisfaction postulated by self-determination theory (Deci and Ryan, 1980). The more students believe that AI is reliable, and beneficial, the more they agree that AI may decrease the threshold for skill acquisition, and give ongoing, efficient support, thus boosting their sense of competence in managing with job uncertainty. At the same time, strong trust leads students to feel that they can independently determine when and how to employ AI tools, satisfying their demand for autonomy. Once these two demands are met, students no longer wait passively for external direction; instead, they actively engage in competency-building activities driven by curiosity, and the need for self-growth, establishing a strong internal drive to overcome AI-induced disruptions. Building on Kong et al. (2023), who related AI trust to employee growth, our results extend the explanatory objective from existing personnel to pre-entry talent and validate the cross-group robustness of the motivating effect of technological trust.

Activated intrinsic motivation directly develops and enhances students' protean career direction. With a high internal growth motives, students tend to view their jobs as sources of self-actualization rather than just as sources of livelihood. They are more likely to step outside organisationally-prescribed careers, create their own learning objectives, look for cross-boundary resources, and judge opportunities in value terms, thus exhibiting the two hallmarks of protean orientation, self-direction and valuing orientation (Briscoe et al., 2006). Moreover, because of the blurring of occupational boundaries with the emergence of AI, high intrinsic motivation students are more likely to view change as opportunity rather than a threat (Zhang et al., 2019). They engage in continual skills development, and frequent shifting of their career direction, and this constant self-guided adjustment is the behavioral manifestation of protean career in a dynamic technological environment. Our results also extend the concluded finding of Rodrigues et al. (2019) that antecedents of career orientations are individual characteristics, with the addition of an important micro-psychological mechanism, motivation.

Intrinsic motivation functions as a psychological mediator that partly explained the positive impact of trust in AI on the protean career. As a result, AI trust not only impacts directly on students' positive expectations about the career path of the AI era, but also, through stimulating a stronger internal drive for self-development, influences indirectly on them to pursue a self-guided, value-based career path. The finding expands the earlier single-level model, in which technology cognition affects career attitudes by hypothesizing and empirically testing the multilevel chain. Technology trust activates motivation first, and motivation then shapes career orientation. It answers the call by Kong et al. (2021) to open the black box between “AI attitudes and career outcomes” and provides a practical lever for the design of university career services. Allowing students to experience both the intrinsic and reliable strength of AI can be a key to unlocking the motivation that drives intrinsic growth and that motivational rush can then translate into the development of protean career.

Job insecurity was also found to significantly moderate the influence of AI trust on intrinsic motivation. This study confirms the underlying logic of conservation of resources theory insofar as resource-loss concern motivates individuals to seek external compensation. When students feel insecure, the predicted loss of career resources raises pressure, and high AI trust is magnified into a dependable avenue for external resource replenishment. Under this apprehensive environment, the satisfying benefits of AI trust on both competence and autonomy requirements are strengthened, so intrinsic motivation is boosted more significantly, and the subsequent mediated pathway through which this motivation translates into protean career is likewise intensified. In contrast, when insecurity is low, the predicted resource loss is minimal, students rely less on the nice-to-have functions of AI, and the motivating effect of trust—and its indirect impact on protean career—is diminished. The conclusion corresponds with Chen et al. (2024), who found that insecurity enhances the impact of external cues on work attitudes in an employee sample. We extend this result to the realm of AI trust and student motivation, indicating that universities should provide varied assistance in AI literacy courses depending on students' level of perceived security.

### 5.2 Theoretical implications

First, we deepen the micro-level mechanism literature relating artificial intelligence to career behavior. Prior research have either studied macro consequences of AI on labor-market structure and skill demand (Frey and Osborne, 2017) or focused on superficial links between AI awareness, usage behavior and career attitudes (Presbitero and Teng-Calleja, 2023). By centring on AI trust and incorporating self-determination theory (Deci and Ryan, 1980), we elucidate how the construct drives protean career. Results suggest that AI trust satisfies students' competence and autonomy demands, hence increasing intrinsic motivation to cope actively with career uncertainty and, in turn, developing protean career. This finding breaks away from the one-way paradigm that attributes career-orientation change to the mere presence of technology, and it reveals a nuanced path in which subjective technology trust motivates orientation formation through motivational activation, offering the field a finer-grained theoretical lens.

Second, we enrich the antecedent literature on protean career direction. Previous research have focused mostly on traditional characteristics such as personality qualities, family support and school socialization (Rodrigues et al., 2019), while rarely including developing technological elements. By incorporating AI trust in the antecedent model, we not only establish a direct positive influence on protean career but also highlight the mediation function of intrinsic motivation. These findings integrate technology trust into the nomological network of protean career, identify a key psychological mechanism through which the career orientation is formed in technology-rich environments, and provide contemporary empirical evidence for Briscoe and Hall (2006) classic argument that protean careers depend on individuals actively transcending external constraints.

Further, we promote a cross-theoretical synthesis of self-determination theory and conservation of resources theory. Self-determination theory focuses on how need satisfaction energizes motivation and shapes behavioral tendencies (Deci and Ryan, 1980), whereas conservation of resources theory centers on the psychological strategies that individuals use to forestall or offset resource loss (Hobfoll, 1989; Hobfoll et al., 2018). Previous research have often implemented each framework in isolation. We integrate them into a single model: self-determination theory clarifies why AI trust, by fulfilling basic psychological needs, triggers intrinsic motivation that transmits the effect to protean career, while conservation of resources theory specifies how job insecurity, as concern over possible loss of career resources, alters reliance on AI trust as a compensatory resource channel and thereby moderates the strength of the AI trust to intrinsic motivation link. This dual-theory approach overcomes the constraints of single-theory accounts and delivers a motivational-mediation plus resource-moderation explanatory scheme that increases our understanding of professional psychology in the AI era and gives an exemplar of multi-theoretical synergy for the discipline.

Finally, we extend the application boundary of COR into technology-driven transition scenarios. Traditionally, this theory has been employed to explain job stress and burnout in organizational settings (Hobfoll, 2002), with a focus on conventional resources such as compensation and advancement prospects (Halbesleben et al., 2014). Our study changes the perspective to AI-induced career resource anxiety, evaluating how employment uncertainty moderates the link between AI trust and intrinsic motivation. We show that technology-triggered fear of resource loss heightens students' belief that AI trust can replenish resources, hence enhancing the motivational benefit of high AI trust. This conclusion verifies the theory's usefulness in technology transition scenarios and broadens its resource domain beyond traditional work assets to the prospective career resources encoded in technology trust. The results enrich the theory's conceptual scope and present an actual example of its utility under new technological settings, thus broadening both the application boundary and explanatory reach of conservation of resources theory.

### 5.3 Practical implications

First, university students should create rational trust in AI to ignite their intrinsic growth motivation. Developing a favorable attitude toward AI is vital for lowering job-search anxiety and helping students cope with the problems of an AI-driven labor market (Li R. et al., 2025). Our findings demonstrate that AI trust meets competence and autonomy demands, and consequently enhances the intrinsic motivation to face professional uncertainty proactively (Deci and Ryan, 1980, 2008). Students are consequently encouraged to obtain hands-on experience with AI tools, such as AI-supported learning applications or AI-based career-planning platforms (Gedrimiene et al., 2024), so that they can witness for themselves how AI increases efficiency, and broadens skills (Chen et al., 2025). Doing so will eventually replace technology-replacement anxieties with the conviction that AI is a career benefit (Kong et al., 2023; Voigt and Strauss, 2025). When employment instability rises, students should convert fear into learning energy, using AI to research upcoming career pathways and to create cross-disciplinary competencies, so transitioning from passive worry to active adaptation.

Second, universities should integrate trust-building and motivation-enhancement tactics into both AI teaching and job coaching. Our findings demonstrate that AI trust not only changes students' professional cognitions but also indirectly molds their protean career through intrinsic motivation. Therefore, AI courses should evolve beyond fundamental tool use and contain modules that link AI to career growth. Case analyses and project-based assignments can give students authentic experience of how AI aids career exploration (Bankins et al., 2024; Presbitero and Teng-Calleja, 2023). In career counseling, customized support should be supplied according to students' level of employment insecurity (Salimi et al., 2025). Students who are extremely nervous should be guided to use AI to unearth career resources and extend their developmental pathways, and low anxiety students should be encouraged to exploit AI to explore numerous job alternatives and further boost their career flexibility (Chui et al., 2022).

Lastly, while our study focuses on university students, its findings may offer preliminary insights for corporate HR practices. Given that students with high AI trust and strong intrinsic motivation are more likely to proactively adapt to technological change, and take control of their career development, it is reasonable to hypothesize that similar dynamics could apply in the workplace. During on-campus recruitment, organizations might consider assessing candidates' attitudes toward AI and their career autonomy, as these factors could potentially predict adaptability and engagement in a rapidly changing technological landscape. However, further research is needed to validate these dynamics in an employment context.

### 5.4 Limitations and future directions

First, the sample is geographically concentrated, and the sampling procedure is non-probabilistic. Data were collected from 80-plus universities in Guangdong Province, leaving other parts of China unrepresented. Convenience, and snowball sampling may create selection bias and reduce generalisability. Future studies should cover colleges countrywide, incorporate multiple institutional kinds, and apply stratified random sampling to eliminate bias, and obtain a more representative picture of AI trust and protean career among Chinese undergraduates.

Second, this study utilized a cross-sectional design, and relied on self-reported data, which introduces potential biases such as common method bias. Although we conducted a single-factor confirmatory factor analysis to assess CMB, and found no significant issues, this method may not fully capture all forms of CMB. The reliance on self-reported measures could also lead to response biases. Furthermore, the directionality between AI trust and intrinsic motivation remains a theoretical assumption. While we used SDT to explain how AI trust enhances intrinsic motivation, it is also plausible that individuals with high intrinsic motivation may be more likely to trust AI. Future research should consider employing longitudinal designs and incorporating objective measures or multi-source data to enhance the robustness of the findings and provide a more comprehensive understanding of the constructs under investigation. Longitudinal studies could also help clarify the temporal precedence and reciprocal relationships between AI trust and intrinsic motivation.

Third, all metrics rely on single-source self-reports, which are prone to social-desirability bias. Future studies might integrate peer ratings, behavioral indicators, and objective career-exploration records, and could deconstruct each component into finer sub-dimensions to understand the micro-processes via which AI trust and protean career evolve.

## 6 Conclusion

In this study, we explored how AI trust influences university students' protean career orientation through the lens of Self-Determination Theory (SDT), and Conservation of Resources (COR) theory. Our findings reveal that AI trust significantly enhances intrinsic motivation, which in turn positively affects protean career orientation. Importantly, job insecurity moderates this relationship, amplifying the effect of AI trust on intrinsic motivation and subsequently on protean career orientation. These results highlight the critical role of AI trust in shaping students' career attitudes in the digital age and underscore the importance of intrinsic motivation as a psychological mechanism.

Overall, our research provides empirical evidence on how technological beliefs can influence career orientations among university students. By integrating SDT and COR theory, we offer a comprehensive framework that explains the interplay between AI trust, intrinsic motivation, and protean career orientation. Future research should consider longitudinal designs and diverse samples to further validate our findings and explore their broader implications.

## Data Availability

The original contributions presented in the study are included in the article/Supplementary material, further inquiries can be directed to the corresponding author.
