# Supplementary material for: AI trust and protean career among university students: the mediating role of intrinsic motivation and the moderating role of job insecurity
Source: Front Psychol. 2026 Jan 23;16:1749655. doi: 10.3389/fpsyg.2025.1749655 (PMC12876185; doi:10.3389/fpsyg.2025.1749655)
Supplement: Supplementary file 1 [file Data_Sheet_1.pdf]

Appendix 1 Measurement

Variables	Items	Code	Reference
AI trust	I have confidence in the use of AI technology	AT1	Kong, H., Yin, Z., Baruch, Y., & Yuan, Y. (2023). The impact of trust in AI on career sustainability: The role of employee–AI collaboration and protean career orientation. Journal of Vocational Behavior, 146, 103928. https://doi.org/10.1016/j.jvb.2023.103928
	I believe AI technology can facilitate routine and trivial tasks through automation	AT2	
	I believe AI technology can be used reliably and consistently without failing	AT3	
	I believe AI technology will consistently operate, providing adequate and efficient results across a wide range of processes	AT4	
	I believe AI adoption will result in the creation of new jobs	AT5	
	I have a positive attitude towards the adoption of AI	AT6	
	I believe AI technology can help develop new skills that will benefit my career development	AT7	
	I have a positive attitude towards its impact on organizational business operations	AT8	
	I believe AI will positively change the way people work within the organization	AT9	
	I believe AI adoption won't reduce the focus on human skills such as creative intellect in my future job	AT10	
	I believe AI adoption will enhance the quality of my future work	AT11	
Intrinsic motivation	To overcome the uncertainty brought about by AI, I hope that my future work will provide me with the opportunity to increase my knowledge and abilities	IM1	Liang, X., Guo, G., Shu, L., Gong, Q., & Luo, P. (2022). Investigating the double-edged sword effect of AI awareness on employee's service innovative behavior. Tourism Management, 92, 104564. https://doi.org/10.1016/j.tourman.2022.104564
	To overcome the uncertainty of AI, I want to know how well I can perform in my future job	IM2	
	Overcoming the uncertainty of AI in my future career brings me joy	IM3	
	Overcoming the uncertainty brought about by AI helps me with self-expression	IM4	
	Regardless of the outcome of overcoming the uncertainty that AI brings, I am content with gaining new experiences	IM5	
	I'm happier when I can set goals for myself to overcome the uncertainty that AI brings in my future career	IM6	
	I am in charge of my own career	SD1	

Protean Career	Ultimately, I depend upon myself to move my career forward	SD2	Briscoe, J. P., Hall, D. T., & Frautschy DeMuth, R. L. (2006). Protean and boundaryless careers: An empirical exploration. Journal of Vocational Behavior, 69(1), 30–47. https://doi.org/10.1016/j.jvb.2005.09.003
	I am responsible for my success or failure in my career	SD3	
	Where my career is concerned, I am very much “my own person	SD4	
	Overall, I have a very independent, self-directed career	SD5	
	I have relied more upon myself than others to find a new job when necessary	SD6	
	Freedom to choose my own career path is one of my most important values	SD7	
	When development opportunities have not been offered by my company, I’ve sought them out on my own.	SD8	
	I follow my own guidance if my company asks me to do something that goes against my values	VD1	
	I have sided with my own values when the company has asked me to do something I don’t agree with	VD2	
	What I think about what is right in my career is more important to me than what my company thinks	VD3	
	It doesn’t matter much to me how other people evaluate the choices I make in my career	VD4	
	I navigate my own career, based upon my personal priorities, as opposed to my employer’s priorities	VD5	
	What’s most important to me is how I feel about my career success, not how other people feel	VD6	
Job Insecurity	I am worried about the possibility of losing my job in the future	JI1	He, C., Teng, R., & Song, J. (2024). Linking employees’ challenge-hindrance appraisals toward AI to service performance: The influences of job crafting, job insecurity and AI knowledge. International Journal of Contemporary Hospitality Management, 36(3), 975–994. https://doi.org/10.1108/IJCHM-07-2022-0848
	My future job feels insecure	JI2	
	I think my future job is likely to change	JI3	
	I don’t think my future job will be permanent	JI4	
	The thought of losing my future job really scares me	JI5	

Appendix 2 Factor loading

item	AI trust	job insecurity	intrinsic motivation	protean career
AT1	0.726			
AT10	0.623			
AT11	0.808			
AT2	0.718			
AT3	0.544			
AT4	0.768			
AT5	0.783			
AT6	0.81			
AT7	0.778			
AT8	0.747			
AT9	0.764			
FJI1		0.808		
FJI2		0.859		
FJI3		0.881		
FJI4		0.738		
FJI5		0.774		
IM1			0.782	
IM2			0.797	
IM3			0.789	
IM4			0.794	
IM5			0.763	
IM6			0.814	
SD1				0.686
SD2				0.766
SD3				0.675
SD4				0.727
SD5				0.762
SD6				0.694
SD7				0.688
SD8				0.707
VD1				0.712
VD2				0.711
VD3				0.66
VD4				0.707
VD5				0.699
VD6				0.681
